# Efficient synthesis of L-lactic acid from glycerol by metabolically engineered *Escherichia coli*

**DOI:** 10.1186/1475-2859-12-7

**Published:** 2013-01-25

**Authors:** Suman Mazumdar, Matthew D Blankschien, James M Clomburg, Ramon Gonzalez

**Affiliations:** 1 Department of Chemical and Biomolecular Engineering, Rice University, 6100 Main Street, MS-362, Houston, TX 77005, USA; 2 Department of Bioengineering, Rice University, Houston TX, USA

**Keywords:** L-lactic acid, Glycerol, Metabolic engineering, *Escherichia coli*

## Abstract

**Background:**

Due to its abundance and low-price, glycerol has become an attractive carbon source for the industrial production of value-added fuels and chemicals. This work reports the engineering of *E. coli* for the efficient conversion of glycerol into L-lactic acid (L-lactate).

**Results:**

*Escherichia coli* strains have previously been metabolically engineered for the microaerobic production of D-lactic acid from glycerol in defined media by disrupting genes that minimize the synthesis of succinate, acetate, and ethanol, and also overexpressing the respiratory route of glycerol dissimilation (GlpK/GlpD). Here, further rounds of rationale design were performed on these strains for the homofermentative production of L-lactate, not normally produced in *E. coli*. Specifically, L-lactate production was enabled by: 1), replacing the native D-lactate specific dehydrogenase with *Streptococcus bovis* L-lactate dehydrogenase (L-LDH), 2) blocking the methylglyoxal bypass pathways to avoid the synthesis of a racemic mixture of D- and L-lactate and prevent the accumulation of toxic intermediate, methylglyoxal, and 3) the native aerobic L-lactate dehydrogenase was blocked to prevent the undesired utilization of L-lactate. The engineered strain produced 50 g/L of L-lactate from 56 g/L of crude glycerol at a yield 93% of the theoretical maximum and with high optical (99.9%) and chemical (97%) purity.

**Conclusions:**

This study demonstrates the efficient conversion of glycerol to L-lactate, a microbial process that had not been reported in the literature prior to our work. The engineered biocatalysts produced L-lactate from crude glycerol in defined minimal salts medium at high chemical and optical purity.

## Background

**Figure 1:**
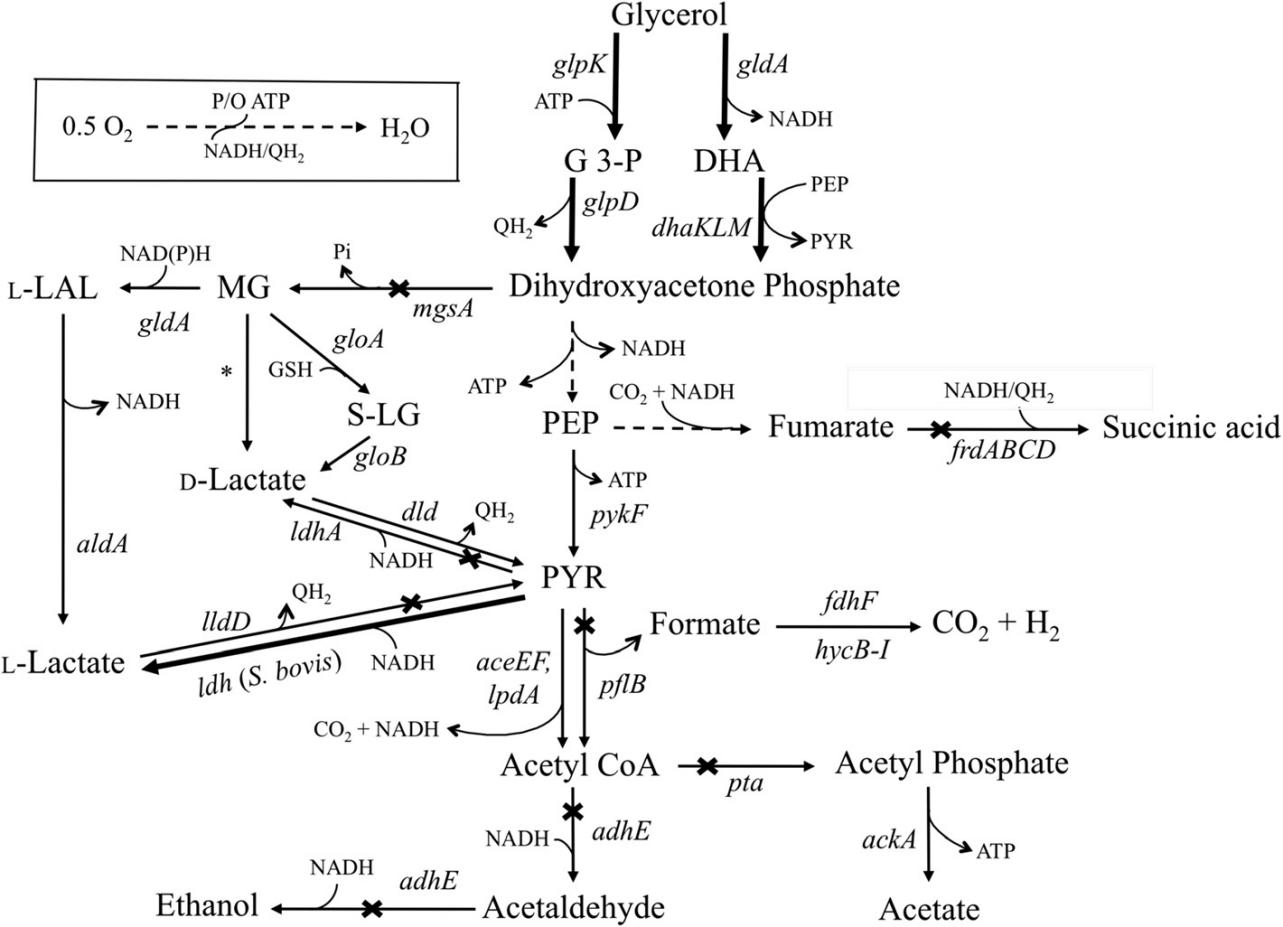
**Pathways involved in the microaerobic utilization of glycerol and the synthesis of fermentation products in native and engineered *E. coli.*** Genetic modifications supporting the metabolic engineering strategies employed in this work are illustrated by thicker lines (overexpression of *E. coli gldA-dhaKLM* and *glpK-glpD* and *S. bovis ldh*) or cross bars (disruption of *pflB*, *pta*, *adhE*, *frdA*, *ldhA*, *mgsA* and *lldD*). Broken lines illustrate multiple steps. Relevant reactions are represented by the names of the gene(s) coding for the corresponding enzymes (*E. coli* genes/enzymes unless otherwise specified in parenthesis): *aceEF-lpdA*, pyruvate dehydrogenase complex; *adhE*, acetaldehyde/alcohol dehydrogenase; *ackA,* acetate kinase; *aldA,* aldehyde dehydrogenase A; *dhaKLM*, dihydroxyacetone kinase; *dld*, respiratory D-lactate dehydrogenase; *fdhF*, formate dehydrogenase, part of fomate hydrogenlyase complex; FrdABCD, fumarate reductase; *gldA*, glycerol dehydrogenase; *gloA*, glyoxalase I; *gloB*, glyoxalase II; *glpD*, aerobic glycerol-3-phosphate dehydrogenase; *glpK*, glycerol kinase; *hycB-I*, hydrogenase 3, part of formate hydrogenlyase complex; *ldh*, fermentative L-lactate dehydrogenase (*S. bovis*); *ldhA*, fermentative D-lactate dehydrogenase; *lldD*, respiratory L-lactate dehydrogenase; *mgsA*, methylglyoxal synthase; *pflB*, pyruvate formate-lyase; pta, phosphate acetyltransferase; *pykF*, pyruvate kinase. Abbreviations: DHA, dihydroxyacetone; DHAP, DHA phosphate; G-3-P, glycerol-3-phosphate; PEP, phosphoenolpyruvate; P_i_, inorganic phosphate; PYR, pyruvate; P/O, amount of ATP produced in the oxidative phosphorylation per pair of electrons transferred through the electron transport system; QH_2_, reduced quinones; S-LG, S-lactoylglutathione; *, glyoxalase III.

Glycerol has recently become an inexpensive and abundant carbon source due to being a byproduct of the biodiesel, oleo-chemical, and bioethanol industries, [1,2]. In addition, future opportunities are available for even larger amounts of glycerol production due to the synthesis and intracellular accumulation of high glycerol concentrations by certain species of algae [3]. Although many microorganisms are able to metabolize glycerol, the use of industrial microbes such as *E. coli* could greatly accelerate the development of platforms to produce fuels and chemicals from this carbon source [4]. We recently reported on the ability of *E. coli* to metabolize glycerol under anaerobic and microaerobic conditions and identified the pathways mediating these metabolic processes (Figure 1) [5-7]. These studies have provided a platform to metabolically engineer *E. coli* for the efficient conversion of glycerol into fuels and industrial chemicals such as ethanol [8-11], hydrogen [11,12], formic acid [11], pyruvic acid [13] and succinic acid [14].

A shared metabolic feature of the anaerobic and microaerobic utilization of glycerol in *E. coli* is the generation of ethanol as the primary product and the negligible production of lactic acid (lactate) [5-7]. However, we have recently reported the engineering of this bacterium for microaerobic production of D-lactate from glycerol in a defined minimal medium [15]. Lactate and its derivatives have many applications in the food, pharmaceutical, and polymer industries [16,17]. An example is polylactic acid, a renewable, biodegradable, and environmentally friendly polymer produced from controlled ratios of D- and L-lactate [18]. Because of the importance of using pure enantiomers in such applications, biological processes have the advantage over chemical means of producing chirally pure lactate from inexpensive media containing only the carbon source and mineral salts [19]. While lactic acid bacteria have been traditionally used in the production of D- and L-lactate from carbohydrate-rich feedstocks, several studies have recently reported alternative biocatalysts such as *E. coli*[16,17], many of which are engineered to produce L-lactate from sugar feedstocks [20-23].

Unlike the aforementioned reports (i.e. use of carbohydrates), our laboratory has focused on the use of glycerol as a carbon source for the production of chemicals with high optical and chemical purity. As such, this manuscript focuses on the metabolic engineering of *E. coli* for the efficient conversion of glycerol to L-lactate, a microbial process that had not been reported prior to our work. The engineered strains hold great promise for the conversion of low-value glycerol streams present in the current biofuels industries to a higher-valued product, L-lactate.

## Results

### Replacement of *E. coli*’s D-lactate specific dehydrogenase with *Streptococcus bovis* L-lactate dehydrogenase and disruption of the methylglyoxal bypass

**Figure 2:**
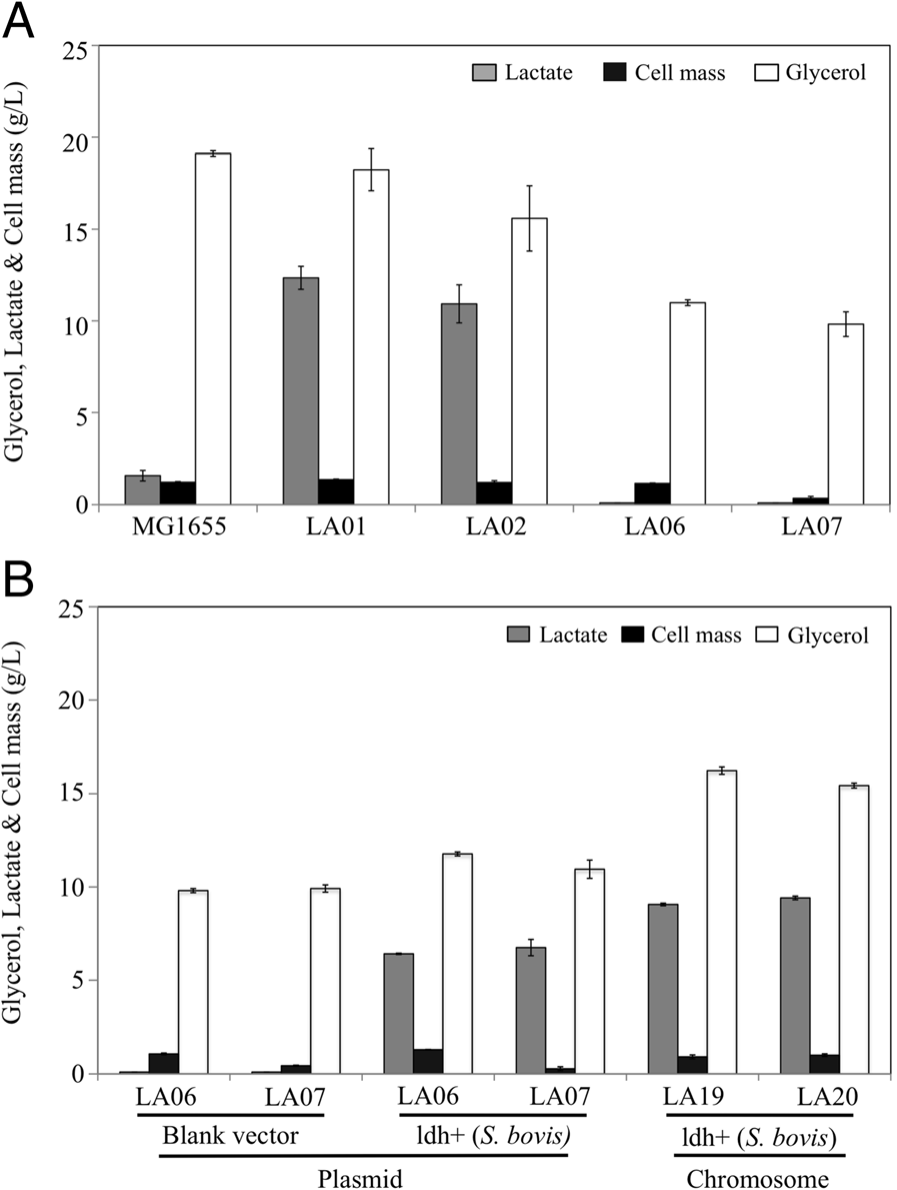
**Cell growth, glycerol utilization, and lactate synthesis in 36-hour shake flasks cultures of wild-type MG1655 and engineered strains.** (**A**) LA01 (Δ*pflB*Δ*frd*A), LA02 (Δ*pta*Δ*adhE*Δ*frdA*), LA06 (Δ*pflB*Δ*frdA*Δ*ldhA*) and LA07 (Δ*pta*Δ*adhE*Δ*frdA*Δ*ldhA*). (**B**) LA06 (pZSblank), LA07 (pZSblank), LA06 (pZSldh), LA07 (pZSldh), LA19 (Δ*pflB*Δ*frdA*Δ*mgsA*Δ*ldhAldh*+), and LA20 (Δ*pta*Δ*adhE*Δ*frdA*Δ*mgsA*Δ*ldhAldh*+). Gene expressions from either plasmid or chromosomal integration are indicated by a “+” next to the corresponding gene(s) or operon(s). Error bars represent standard deviations for triplicate measurements.

*E. coli* strains LA01 (Δ*pflB*Δ*frdA)* and LA02 (Δ*pta*Δ*adhE*Δ*frdA*) are initial platforms developed to demonstrate the microaerobic production of optically pure D-lactate in mineral salts medium using glycerol [15]. LA01 contains a deletion in *pflB* (pyruvate formate lyase, PFL), which minimizes the production of ethanol and acetate due to the fact that PFL is the primary route for pyruvate conversion to acetyl-CoA during the microaerobic utilization of glycerol [5]. LA01 also possess an *frdA* deletion (component of fumarate reductase) to reduce the synthesis of succinate (Figure 1). LA02, on the other hand, is a triple mutant in which the synthesis of ethanol (Δ*adhE*), acetate (Δ*pta*), and fumarate (Δ*frdA*) have been blocked through respective gene deletions directly involved with their synthesis (Figure 1). Both strains produced D-lactate as the primary product of glycerol metabolism (Figure 2A and Table 1, rates of 0.34 and 0.30 g/L/h, respectively) under microaerobic conditions (Additional file 1: Figure S1).

**Table 1:** **Glycerol consumption, product synthesis, and carbon recovery in cell mass and fermentation products during the microaerobic utilization of glycerol in minimal medium by wild-type and engineered strains**^**a**^

**Strain**	**Glycerol consumed (g/L)**	**Product synthesized (g/L)**						**Carbon recovery**^**b**^	
		**Acetate**	**Succinate**	**Ethanol**	**Lactate**	**Pyruvate**	**Biomass**	**Products**	**Overall**
Wild-type strain									
MG1655	19.1 (0.1)	2.9 (0.1)	2.4 (0.0)	2.1 (0.1)	1.6 (0.3)	0.0 (0.0)	1.2 (0.0)	67.4 (1.4)	75.4 (1.6)
Strains engineered for the production of D-lactate												
LA01	18.2 (1.1)	0.6 (0.1)	0.0 (0.0)	0.0 (0.0)	12.3 (0.6)	0.0 (0.0)	1.4 (0.0)	74.9 (3.4)	84.3 (3.7)			
LA02	15.6 (1.8)	0.6 (0.0)	0.1 (0.0)	0.0 (0.0)	10.9 (1.0)	0.0 (0.0)	1.2 (0.1)	77.6 (2.8)	87.4 (3.4)			
Strains engineered for the production of L-lactate												
LA06	11.0 (0.2)	2.0 (0.1)	0.1 (0.0)	0.0 (0.0)	0.1 (0.0)	0.0 (0.0)	1.2 (0.0)	27.8 (0.5)	67.2 (0.9)			
LA07	9.8 (0.7)	1.2 (0.4)	0.2 (0.1)	0.0 (0.0)	0.1 (0.0)	0.0 (0.0)	0.35 (0.1)	20.7 (5.5)	34.0 (2.2)			
LA06 (pZS)	9.8 (0.1)	1.8 (0.1)	0.1 (0.0)	0.0 (0.0)	0.1 (0.0)	0.0 (0.0)	1.1 (0.1)	29.2 (1.6)	70.0 (0.1)			
LA07 (pZS)	9.9 (0.2)	1.5 (0.0)	0.3 (0.0)	0.0 (0.0)	0.1 (0.0)	0.0 (0.0)	0.4 (0.0)	30.1 (0.6)	46.7 (2.0)			
LA06 (pZSldh)	11.8 (0.1)	0.6 (0.0)	0.1 (0.0)	0.0 (0.0)	6.4 (0.0)	0.0 (0.0)	1.3 (0.0)	64.9 (0.2)	78.6 (0.2)			
LA07 (pZSldh)	10.9 (0.5)	0.2 (0.0)	0.1 (0.0)	0.0 (0.0)	6.7 (0.4)	0.0 (0.0)	0.3 (0.1)	65.7 (2.9)	70.4 (1.9)			
LA19	16.2 (0.2)	1.0 (0.0)	0.0 (0.0)	0.0 (0.0)	9.1 (0.1)	0.0 (0.0)	0.9 (0.1)	67.4 (0.2)	74.5 (1.1)			
LA20	15.4 (0.1)	0.4 (0.0)	0.2 (0.0)	0.0 (0.0)	9.4 (0.1)	0.0 (0.0)	1.0 (0.1)	67.7 (0.2)	75.8 (0.5)			
LA19 (pZSKLMgldA)	16.6 (0.6)	1.0 (0.0)	0.0 (0.0)	0.2 (0.0)	10.9 (0.4)	0.0 (0.0)	0.4 (0.0)	79.0 (0.4)	82.1 (0.9)			
LA19 (pZSglpKglpD)	16.8 (0.9)	0.7 (0.1)	0.0 (0.0)	0.0 (0.0)	11.7 (1.2)	0.0 (0.0)	0.8 (0.0)	79.7 (3.9)	85.9 (3.8)			
LA19 (pZSldh)	13.2 (1.6)	0.6 (0.1)	0.0 (0.0)	0.0 (0.0)	7.4 (1.3)	0.0 (0.0)	0.7 (0.1)	65.6 (4.6)	74.4 (3.4)			
LA20 (pZSKLMgldA)	15.2 (0.3)	0.4 (0.1)	0.1 (0.0)	0.0 (0.0)	10.1 (0.5)	0.0 (0.0)	0.9 (0.2)	72.9 (1.5)	80.3 (3.1)			
LA20 (pZSglpKglpD)	18.6 (0.4)	0.6 (0.0)	0.2 (0.0)	0.0 (0.0)	13.7 (0.7)	0.0 (0.0)	0.8 (0.0)	76.4 (1.7)	82.8 (1.6)			
LA20 (pZSldh)	15.3 (0.2)	0.2 (0.0)	0.2 (0.1)	0.0 (0.0)	10.0 (0.1)	0.0 (0.0)	0.9 (0.0)	70.5 (1.6)	77.6 (2.0)			
LA20 (pZSglpK.glpD)^c^	41.0 (0.0)	1.0 (0.0)	0.7 (0.0)	0.0 (0.0)	32.6 (0.1)	0.0 (0.0)	1.1 (0.0)	86.4 (0.3)	89.6 (0.3)			
LA20ΔlldD (pZSglpKglpD)^c^	41.6 (0.0)	0.8 (0.0)	0.6 (0.0)	0.0 (0.0)	34.7 (0.0)	0.0 (0.0)	1.6 (0.0)	90.3 (0.0)	95.0 (0.1)			
LA20ΔlldD (pZSglpKglpD)^d^	40.4 (0.0)	0.9 (0.0)	0.9 (0.0)	0.0 (0.0)	32.8 (0.1)	0.0 (0.0)	1.1 (0.0)	88.4 (0.2)	91.9 (0.1)			
LA20ΔlldD (pZSglpKglpD)^e^	57.2 (0.0)	1.6 (0.0)	1.2 (0.0)	0.0 (0.0)	50.1 (0.0)	0.0 (0.0)	2.0 (0.0)	95.2 (0.1)	99.4 (0.7)			

^a^ Data represent the average of three samples (standard deviations shown in parenthesis) taken from 36-hour shake flask cultures grown on minimal medium supplemented with 20 g/L of glycerol, unless otherwise specified.

^b^ Carbon recovery is expressed as the percent mol of carbon in product, including biomass, per mol of carbon in glycerol consumed. The column “product” shows the total recovery of carbon in products, assuming that moles of acetate plus moles of ethanol equals moles of 1-C compounds (formate plus CO2) generated by the dissimilation of pyruvate. The column “overall” shows the overall carbon recovery, including products and biomass.

^c^ Cultures in which 40 g/L of glycerol was used and samples were taken at 72 hours (all glycerol was consumed).

^d^ A culture in which 40 g/L of crude glycerol derived from biodiesel production was used and samples were taken at 72 hours (all glycerol was consumed).

^e^ A culture in which 60 g/L of crude glycerol (40 g/L initially present and 20 g/L added at 48 hours) was used and samples were taken at 84 hours (~ 57 g/L of glycerol were consumed).

**Figure 3:**
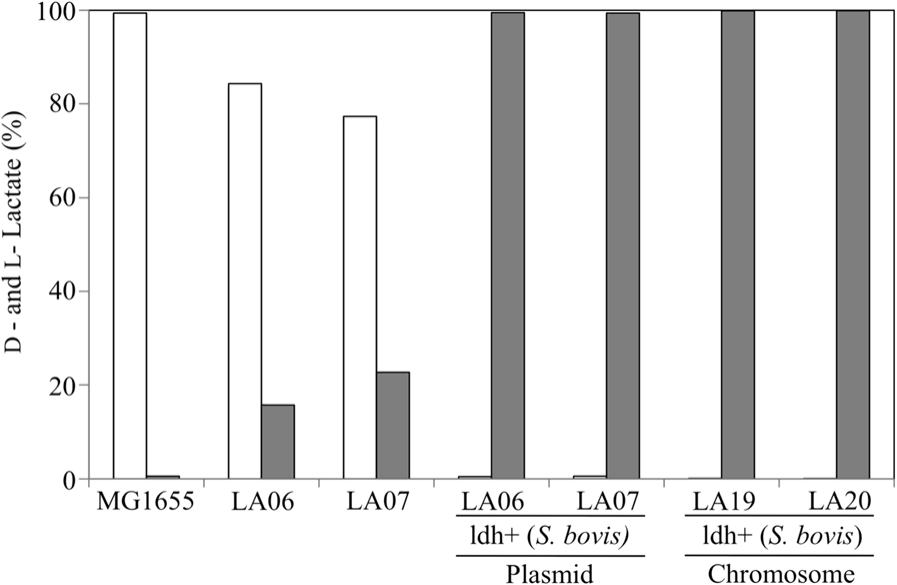
**Enantiomeric composition determined enzymatically of lactate produced by wild-type MG1655 and engineered strains LA06, LA07, LA06 (pZSldh), LA07 (pZSldh), LA19, and LA20.** The percentage of each enantiomer in the mixture is shown: D-lactate (white bar) and L-lactate (gray bar).

To initiate the metabolic engineering of these previous LA01 and LA02 platforms for the production of L-lactate, the fermentative *E. coli* D-lactate dehydrogenase (D-LDH) was eliminated, resulting in strains LA06 (LA01Δ*ldh*A) and LA07 (LA02Δ*ldhA*). As expected, very small amounts of lactate (final titers of ~0.1 g/liter in both cases) were detected in the fermentation broth of strains LA06 and LA07 (Figure 2A), demonstrating that D-LDH (*ldhA*) is the primary route of lactate production in these *E. coli* platforms. The lactate produced was a racemic mixture of D- and L-lactate (Figure 3), suggesting their production through the MG detoxification pathways [24-27] (Figure 1). The ability of strains LA06 and LA07 to produce large amounts of lactate was restored by the presence of plasmid pZSldh which expresses the L-lactate dehydrogenase (L-LDH) from *S. bovis* (Figure 2B and Table 1, rates of ~0.19 g/L/h for both). The enantiomeric purity of the produced L-lactate was high in both cases (~99.5%, Figure 3).

Given the above results, the S. *bovis ldh* gene was chromosomally integrated in strains LA01 and LA02 and the *E. coli mgsA* gene was simultaneously deleted to avoid any production of D-lactate through the MG bypass. The *ldhA* locus was chosen as the integration site because the levels of expression of D-LDH from this promoter in LA01 and LA02 were shown to support efficient production of D-lactate [15] and could presumably support L-lactate production as well. The resulting LA19 (Δ*pflB*Δ*frdA*Δ*mgsA*Δ*ldhAldh+*) and LA20 (Δ*pta*Δ*adhE*Δ*frdA*Δ*mgsA*Δ*ldhAldh*+) strains performed well, producing ~9 g/liter of L-lactate in 36 hours (~0.25 g/L/h) (Figure 2B). However, strain LA20 exhibited a slightly better lactate yield (0.61 g/g glycerol compared to 0.56 g/g glycerol in LA19) and lower acetate production (discussed below) (Figure 2B and Table 1). As postulated, the expression of *ldh* from the *ldhA* promoter resulted in L-LDH activity levels similar to those reported for D-LDH in strains LA01 and LA02: i.e. 0.136±0.005 μmol/mg protein/min in LA20 (Table 2) compared to 0.082±0.005 μmol/mg protein/min in LA01 [15]. Finally, the lactate produced by LA20 was 99.9% L-lactate, with similar enantiomeric purity found for strain LA19 (Figure 3).

**Table 2:** Functional characterization of constructs used in the overexpression of glycerol utilization and L-lactate synthesis enzymes

	**Activity (μmol/mg protein/min)**^**a**^	
Enzyme tested	LA20 (Control)^b^	LA20 (Overexpressed)^c^
Glycerol kinase	0.187 ± 0.005	0.669 ± 0.004
Aerobic glycerol-3-phosphate dehydrogenase	0.017 ± 0.001	0.027 ± 0.002
Glycerol dehydrogenase	0.049 ± 0.002	0.39 ± 0.02
Dihydroxyacetone kinase	0.005 ± 0.001	0.019 ± 0.002
L-Lactate dehydrogenase	0.136 ± 0.005	0.68 ± 0.06

^a^ All activities were measured as described in Materials and Methods and values are reported as average ± standard deviation for triplicate assays.

^b^ Activities measured in strain LA20 containing the blank vector.

^c^ Activities measured in strain LA20 containing a plasmid overexpressing the specified enzyme: i.e. pZSKLMgldA for glycerol dehydrogenase and dihydroxyacetone kinase, pZSglpKglpD for glycerol kinase and aerobic glycerol-3-phosphate dehydrogenase, and pZSldh for S. bovis L-lactate dehydrogenase.

Reported values are from 36-hour shake flask cultures.

### Overexpression of glycerol-utilization and L-lactate synthesis pathways and elimination of the endogenous pathway for L-lactate utilization

Although strains LA19 and LA20 produced L-lactate at high chemical and chiral purity, the kinetics of glycerol utilization and lactate synthesis, including lactate titer and yield, were inferior to that of the LA01 and LA02 parental strains (compare panels A and B in Figure 2; Table 1). Since we have previously shown that the conversion of glycerol to D-lactate can be accelerated by amplifying either glycerol-utilization or lactate-synthesis pathways [15], we investigated whether similar strategies could be implemented in the production of L-lactate.

Two primary routes can mediate the conversion of glycerol to the common intermediate, dihydroxyacetone (DHAP) under microaerobic conditions [5] (Figure 1). A fermentative pathway converts glycerol to dihydroxyacetone (DHA) via glycerol dehydrogenase (*gldA*) and then to DHAP through the action of DHA kinase (*dhaKLM*). The alternative route is a respiratory/aerobic pathway composed of the enzymes glycerol kinase (*glpK*) and glycerol-3-phosphate (G3P) dehydrogenase (*glpD*) which mediates the conversion of glycerol to G3P and subsequently to DHAP, respectively. Overexpression of either one of the pathways in LA19 (0.3 and 0.33 g/L/h for fermentative and respiratory routes, respectively) and LA20 (0.28 and 0.38 g/L/h for fermentative and respiratory routes, respectively) led to faster utilization of glycerol and L-lactate synthesis, although the respiratory pathway led to higher L-lactate titers and yields (Table 1, 2). Coupling of glycerol-3-phosphate oxidation and oxygen reduction via the quinine pools [28,29] likely results in the preferential synthesis of L-lactate due to the fact that the overall conversion of glycerol to lactate becomes a redox balanced pathway. In addition, ATP would be generated by both substrate-level phosphorylation and the respiratory chain (see Figure 1 and Discussion).

Another limiting factor for lactate synthesis in strains LA19 and LA20 could be insufficient levels of L-lactate dehydrogenase due to less expression from the chromosomal copy of *S. bovis ldh* as opposed to plasmid overexpression. Thus, expression of *ldh* from a plasmid could alleviate this limitation and lead to an increase in the fraction of carbon diverted towards the synthesis of L-lactate (increasing L-lactate yield) and/or the flux of the glycerol-to- L-lactate pathway (increasing the rate of L-lactate production). This strategy led to a slight increase in the production of L-lactate in LA20 [pZSldh] (Table 1), which was arguably caused by the 5-fold increase in the activity of L-LDH (Table 2). In contrast, overexpression of L-LDH had no beneficial effect on lactate production or glycerol utilization in strain LA19 [pZSldh] (Table 1). Thus, plasmid overexpression of *S. bovis ldh* was not deemed more beneficial than that of the chromosomal copy and not explored further.

Of note, strain LA19 (Δ*pflB*Δ*frdA*Δ*mgsA*Δ*ldhAldh*+) and its parent and derivatives produced much higher concentrations of acetate than that observed in the LA20 (Δ*pta*Δ*adhE*Δ*frdA*Δ*mgsA*Δ*ldhAldh*+) strain and its parent and derivatives (Table 1). While PFL is the primary route for pyruvate conversion to acetyl-CoA during the microaerobic utilization of glycerol, low levels of acetyl-CoA and subsequently acetate could still be formed in the LA19 lineage via leakiness of the primarily aerobic pyruvate dehydrogenase complex (*aceEF* and *lpdA*, Figure 1) [5]. As acetate formation in the LA20 lineage is directly blocked by a *pta* deletion, lower acetate levels would be expected. Increased acetate formation in the LA19 lineage could also explain the differential growth observed between LA06 (i.e. Δ*pflB*, *pta*^+^ etc.) and LA07 (i.e. *pflB*^+^, Δ*pta*). As these strains are deleted for endogenous *ldhA*, they cannot readily synthesize any common fermentative product to achieve redox balance and allow continued ATP production. In this context, the small increases in acetate levels seen in the LA06 would be critical for growth as acetate formation results in 2 ATP molecules per glycerol consumed via substrate level phosphorylation (Figure 1). Only when the higher glycerol utilization and subsequent L-lactate synthesis were achieved with the more optimal expression of *S. bovis ldh* from the chromosome (as opposed to from a plasmid) did the growth between the LA19 and LA20 and direct derivatives become similar (Figure 2 and Table 1). As the LA20 lineage was deemed better than that of LA19 and previous work by us has shown no additional benefit of using the *pflB* deletion in conjunction with just directly blocking the competing fermentative products (data not shown) we choose to use LA20 as our platform for further metabolic engineering.

**Figure 4:**
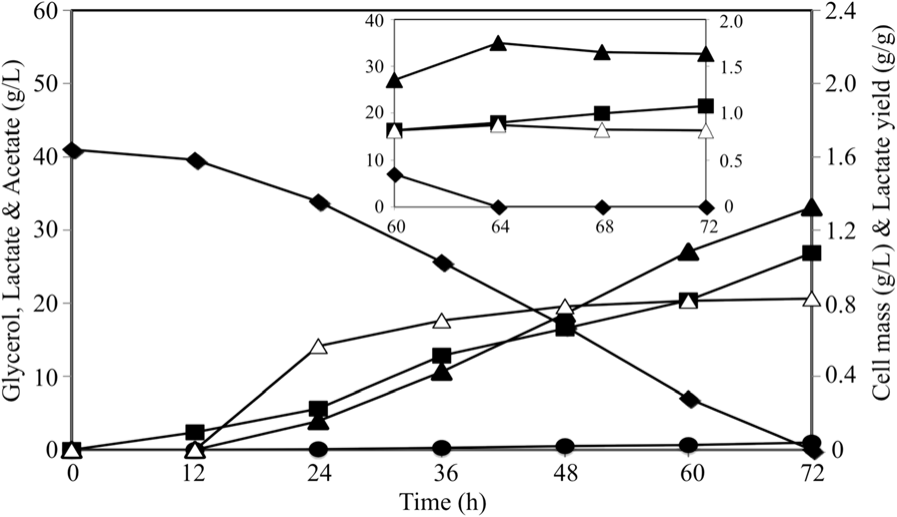
**Kinetics of lactate synthesis by strain LA20 (pZSglpKglpD) in shake flasks containing minimal medium with 40 g/L of glycerol.** Data for concentration of cells (∎), glycerol (♦), lactate (▲) and acetate (●), along with lactate yield (∆), are shown. Coefficients of variation (i.e. standard deviations/average × 100) were below 5% in all cases. The inset shows a high-resolution data set for late stages of cultivation: symbols and axis titles are as specified for the main figure.

Overall, the best performance was observed when the respiratory glycerol-utilization pathway was overexpressed in the LA20 platform (Table 1, and see rates in text above). Using 20 g/L of glycerol, LA20 (pZSglpKglpD) produced 13.7 g/L of L-lactate (0.38 g/L/h) at a yield of 0.74 g L-lactate/g glycerol. Given these results, we further examined the production of L-lactate by LA20 (pZSglpKglpD) in the presence of a higher concentration of glycerol. Starting now with 40 g/L of glycerol, this strain produced about 33 g/liter of L-lactate in less than 72 hours (0.46 g/L/h) at a yield of 0.82 g L-lactate/g glycerol (Figure 4 and Table 1). Besides L-lactate, only small amounts of acetate were found in the culture medium, demonstrating the homolactic nature of the fermentation (Table 1). However, a closer examination of the dynamics of cell growth, glycerol consumption and product synthesis at the late stages of the fermentation revealed interesting behavior: the cultures never reached stationary phase, even when all glycerol was consumed, and a decrease in both L-lactate concentration and yield occurred (Figure 4, Inset). Based on these observations, the accumulation of large amounts of L-lactate in the medium was hypothesized to trigger its consumption by the respiratory L-lactate dehydrogenase (*lldD*), which can catalyze the oxidation of L-lactate to pyruvate [30]. Deletion of the *lldD* gene in strain LA20 and overexpression of the GlpK-GlpD pathway resulted in a clear stationary phase following glycerol depletion from the medium and no decrease in lactate yield or concentration was observed (data not shown). This strain, named LA20 Δ*lldD* (pZSglpKglpD), produced 35.1g/liter of L-lactate from 41.6 g/liter of glycerol in about 64 hours (0.55 g/L/h) with an overall product yield of 0.86 g L-lactate/g of glycerol, which clearly surpasses the performance of its parent LA20 (pZSglpKglpD) (Table 1).

### Production of L-lactate at high concentrations from crude glycerol

**Figure 5:**
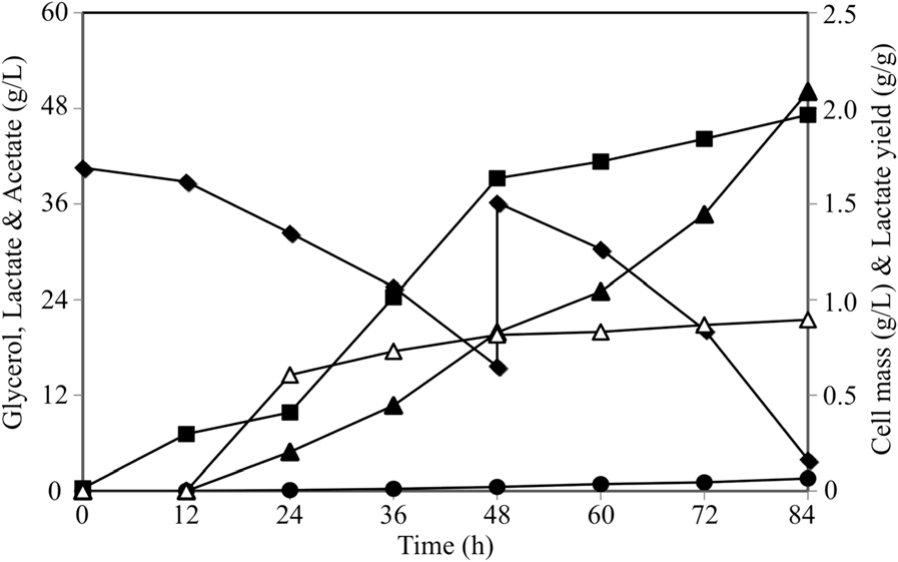
**Production of lactate by strain LA20Δ*lldD *(pZSglpKglpD) in a minimal medium containing crude glycerol.** A fermentation profile using 60 g/liter glycerol of crude glycerol (40 g/liter in the initial medium followed by a 20 g/liter addition at 48 hours) is shown. Data for concentration of cells (∎), glycerol (♦), lactate (▲), and acetate (●), along with lactate yield (∆), are shown. Coefficients of variation (i.e. standard deviations/average × 100) were below 5% in all cases.

The use of an industrial medium containing crude glycerol generated as a by-product in the biodiesel industry is of great relevance for the biocatalyst developed in this work. Engineered strains performed very well when crude glycerol was used as a carbon source. Glycerol consumption and L-lactate synthesis by strain LA20 Δ*lldD* (pZSglpKglpD) using 40 g/liter crude glycerol were similar to those reported for the consumption of pure glycerol (Table 1). To better assess the potential of this process, an experiment with even higher concentrations of crude glycerol was conducted. Under these conditions, strain LA20 Δ*lldD* (pZSglpKglpD) produced more than 50 g/liter of L-lactate in 84 hours at a yield of 0.90 g L-lactate/g glycerol (Figure 5). Maximum and average volumetric rates of L-lactate production of 1.3 g/L/h and ~0.6 g/liter/h were respectively, achieved.

## Discussion

L-lactate production from sugars can be achieved using native lactic acid bacteria but are constrained by the requirements for complex nutrients and exhibit limitations in both product selectivity and enantiomeric purity [16,17]. To overcome these issues, bacteria and yeasts have been engineered to produce L-lactate as the primary product of carbohydrate fermentations [16,17,20-23]. However, the production of L-lactate from glycerol has not been reported. The work conducted here focuses on the metabolic engineering of *E. coli* for the microaerobic production of L-lactate, at high chemical (97%) and optical (99.9%) purities, from glycerol in defined minimal salts medium. Using LA20Δ*lldD* [pZSglpKglpD], 50 g/liter of L-lactate were produced in 84 hours at a yield of 0.90 g L-lactate/g glycerol (Figure 5) with a yield close to 93% of the theoretical maximum (0.967 wt/wt) when calculated from equation 2 below. Besides providing a high yield and productivity, the resulting biocatalyst can also utilize crude glycerol as carbon source, which has become an abundant and inexpensive feedstock due to being a by-product of the current biofuel industries [31].

While L-lactate is not a native product of glycerol metabolism in *E. coli*, its homologous production could be achieved through a two-step pathway that converts methylglyoxal (MG, an intermediate in the MG bypass) to L-lactaldehyde (L-LAL) and then to L-lactate [24-27] (Figure 1). However, the synthesis of L-lactate through these pathways is not desirable due the existence of several native MG detoxification pathways in *E. coli* that can lead to the production of both D- and L-lactate (Figure 1) [25-27], compromising the enantiomeric purity of the product (Figure 3). In addition, MG is a very toxic metabolite whose accumulation could severely impair metabolism and lead to cell death [25,27]. Finally, the synthesis of L-lactate through the MG route is energy inefficient, as no ATP is generated in the conversion of dihydroxyacetone phosphate (DHAP) to L-lactate (Figure 1). Since the generation of DHAP from glycerol consumes one ATP equivalent (in the form of ATP or PEP), the overall conversion of glycerol to L-lactate through this route would lead to the net consumption of one ATP equivalent per each molecule of L-lactate produced (Figure 1). This route, summarized in the equation 1 below by assuming glycerol dissimilation through the GlpK-GlpD pathway (Figure 1), would also generate one net reducing equivalent per L-lactate synthesized:

(1) $$\mathrm{Glycerol}+\mathrm{ATP}+Q\to L-\mathrm{lactate}+\mathrm{ADP}+P+QH_{2}$$

A more attractive alternative to the above MG route that we chose to utilize in this study is the production of L-lactate utilizing enzymes from the later stages of Embden-Meyerhof-Parnas pathway which would avoid the aforementioned disadvantages. In this scenario (see Figure 1), the overall conversion of glycerol to lactate would lead to the net generation of one ATP (via substrate-level phosphorylation) and one reducing equivalent per each molecule of L-lactate produced, as shown in the equation 2 below:

(2) $$\mathrm{Glycerol}+\mathrm{ADP}+P+Q\to L-\mathrm{lactate}+\mathrm{ATP}+QH_{2}$$

Since the GlpK-GlpD pathway mediates glycerol dissimilation in the engineered strain and microaerobic conditions were used (see Supplemental Materials and Additional file 1: Figure S1), coupling the overall transfer of electrons from glycerol-3-phosphate to oxygen (combination of GlpD and CyoABCD) [28,29] can be achieved. This coupling could theoretically generate 1.14 ATPs via oxidative phosphorylation per molecule of glycerol dissimilated [32]. However, given the lower experimental values typically observed [33], the synthesis of 1 ATP per glycerol-3-phosphate molecule oxidized is probably a more reasonable assumption and is detailed in equation III below:

(3) $$QH_{2}+0.5O_{2}\to H_{2}O+\mathrm{ATP}$$

From equations 2 and 3 it then becomes apparent that the synthesis of L-lactate from glycerol can generate up to two molecules of ATP per molecule of L-lactate produced. Overall, this high ATP yield explains why using the later stages of the Embden-Meyerhof-Parnas pathway with overexpression of the respiratory GlpK-GlpD pathway in LA20 was beneficial (as opposed to the use of the MG route).

Given the beneficial nature of the engineered glycerol-to-L-lactate pathway (i.e. redox balanced and ATP generating), we expect that the future use of metabolic evolution approaches will lead to the selection of even more productive biocatalysts. Similar techniques have been successfully implemented in *E. coli* for the efficient production of biofuels and other products [34-36]. Process-based modifications such as fed-batch cultivations and high-density cultures are also envisioned to further improve the volumetric rates of L-lactate production.

## Conclusions

The present study demonstrates the conversion of glycerol to L-lactate, a microbial process that had not been reported to date prior to this study. The engineered biocatalyst produced L-lactate from glycerol in a defined minimal salts medium at high chemical and optical purity. The high yields and productivities achieved with the use of crude glycerol as carbon source, which has become an abundant and inexpensive feedstock, demonstrate that low-value glycerol streams from the current biofuels industries can be efficiently converted to higher value products such as L-lactate.

## Methods

### Strains, plasmids, and genetic methods

Strains LA01 and LA02 (see Table 3 for genotype) were used as hosts to engineer the production of L-lactate. Gene knockouts were introduced by P1 phage transduction [11,37]. Single gene knockout mutants from the National BioResource Project (NIG, Japan) were used as donors of specific mutations [38]. Replacement of native *ldhA* (encoding D-lactate dehydrogenase) with *Streptococcus bovis ldh* (encoding L-lactate dehydrogenase) was achieved via a previously reported method for allele replacement using the *sacB*-containing pWM91 suicide vector [39]. Plasmid pVALDH1 [40], kindly provided by Dr. T. R. Whitehead (National Center for Agricultural Utilization Research, U.S. Department of Agriculture, Agricultural Research Service, Peoria, IL) was the source of the *ldh* gene and primers c-*ldh* (Table 3) were used for cloning purposes. All chromosomal disruptions and replacements were confirmed by polymerase chain reaction using the “verification” primers shown in Table 3. The disruption of multiple genes in a common host was achieved as previously described [11]. All resulting strains, along with primers and plasmids used in this study, are listed in Table 3.

**Table 3:** Strains, plasmids and primers used in this study

**Strain/ Plasmid/Primer**	**Description/Genotype/Sequence**	**Source**
Strains^a^		
MG1655	F- λ- *ilvG*- *rfb*-50 *rph*-1	[41]
LA01	MG1655 Δ*pflB*::FRT Δ*frdA*::FRT-*Kan*-FRT; sequential deletion of *pflB* and *frdA* in MG1655	[15]
LA02	MG1655 Δ*pta*::FRT Δ*adhE*::FRT Δ*frdA*::FRT-*Kan*-FRT; sequential deletion of *pta*, *adhE* and *frdA* in MG1655	[15]
LA06	LA01 Δ*ldhA*::FRT-*Kan*-FRT	This study
LA07	LA02 Δ*ldhA*::FRT-*Kan*-FRT	This study
LA19	LA01 Δ*mgsA*::FRT Δ*ldhA*::ldh	This study
LA20	LA02 Δ*mgsA*::FRT Δ*ldhA*::ldh	This study
LA19Δ*lldD*	LA01 Δ*mgsA*::FRT Δ*ldhA*::ldh Δ*lldD*::FRT	This study
LA20Δ*lldD*	LA02 Δ*mgsA*::FRT Δ*ldhA*::ldh Δ*lldD*::FRT	This study
Plasmids		
pCP20	reppSC101ts ApR CmR cI857 l PR flp+	[42]
pZSblank	Blank plasmid created by removing *C. freundii dhaKL* from pZSKLcf and self-ligating the plasmid (tetR, oriR SC101*, *cat*)	[11]
pWM91	f1(+) ori *lacZ*α of pBluescript II (SK+) mobRP4, oriR6K,*SacB* and AmpR	[39]
pZSKLMgldA	*E. coli dhaKLM* and *gldA* under control of PLtetO-1 (tetR, oriR SC101*, *cat*)	[11]
pZSglpKglpD	*E. coli glpK* and *glpD* under control of P_LtetO-1_ (tetR, oriR SC101*, *cat*)	[15]
pZSldh	*S. bovis ldh* under control of P_LtetO-1_ (tetR, oriR SC101*, *cat*)	This study
Primers ^b^		
v-*pflB*	aaatccacttaagaaggtaggtgtcgtggagcctttattgtac	This study
v-*frdA*	taccctgaagtacgtggctgaggtagttgcgtcataaggc	This study
v-*pta*	ccaaccaacgaagaactggttagcgcaaatattcccttgc	This study
v-*adhE*	cgagcagatgatttactaaaaaagatcggcattgcccagaagg	This study
v-*lldD*	cagtttcgatattctggaagcgacagattcatgctgcg	This study
v-*ldhA*	gcttaaatgtgattcaacatcactggagaatagaggatgaaaggtcattg	This study
c-*ldh*	gacggtaccatgactgcaactaaacaacacaaaaaaggtacggatccttagtttttgcaagcagaagcgaattc	This study
r1-*ldh*	tgctgtacatgactgcaactaaacaacactcgtgtacattagtttttgcaagcagaagc	This study
r2-*ldh*	cttacggtcaattgttgacgcgtcaacaattgaccgtaag	This study

^a^ Deletions were moved into each strain in the order they appear in the “description” column.

^b^ “v”, “c” and “r” indicate the primer sequences (5’ to 3’) that were used for verification purposes (“v”) during gene disruptions, cloning (“c”) of S. bovis ldh, and chromosomal replacement (“r”) of *E. coli ldhA* with *S. bovis ldh* (“r”). “r1” and “r2” were used to confirm the presence *S. bovis ldh* in the *E. coli* chromosome (“r1”) along wit its presence in the *ldhA* locus (“r2”). The forward sequence follows the reverse sequence in each case. Genes or operons manipulated are apparent from primer names.

Gene overexpression was achieved by cloning the desired gene(s) in a low-copy vector as previously reported [15] (Table 3). Plasmid pZSldh was constructed as follows. The *ldh* gene from *S. bovis* was PCR amplified from plasmid pVALDH1 [40] using c-*ldh* primers (Table 3). The resulting PCR product was cloned within the *Kpn*I and *Mlu*I sites of pZSKLMgldA [11] using In-Fusion PCR cloning (Clontech Laboratories, Inc., Mountain View, CA). PCR was performed using Pfu turbo DNA polymerase (Stratagene, CA, USA) under standard conditions described by the supplier. The ligated products were used to transform *E. coli* DH5αT1 (Invitrogen, Carlsbad, CA). Positive clones were screened by plasmid isolation and restriction digestion.

Standard recombinant DNA procedures were used for gene cloning, plasmid isolation, and electroporation. Manufacturer protocols and standard methods [37,43] were followed for DNA purification (Qiagen, Valencia, CA), restriction endonuclease digestion (New England Biolabs, Ipswich, MA), and DNA amplification (Stratagene, La Jolla, CA and Invitrogen, Carlsbad, CA). The strains were kept in 32.5% glycerol stocks at −80°C. Plates were prepared using LB medium containing 1.5% agar, and appropriate antibiotics were included at the following concentrations: ampicillin (50 μg/ml), kanamycin (50 μg/ml), chloramphenicol (12.5 μg/ml), and tetracycline (3.33 μg/ml).

### Culture medium and cultivation conditions

Unless otherwise stated, all fermentations were conducted using the minimal medium designed by Neidhardt *et al.*[44] with Na_2_HPO_4_ in place of K_2_HPO_4_ and supplemented with 20 g/liter glycerol (unless otherwise specified), 5 μM sodium selenite, 3.96 mM Na_2_HPO_4_, 5 mM (NH_4_)_2_SO_4_, and 30 mM NH_4_Cl. Chemicals were obtained from Fisher Scientific (Pittsburgh, PA) and Sigma-Aldrich Co. (St Louis, MO), except crude glycerol, which was provided by Renewable Energy Group, Inc. (Ames, IA). Crude glycerol had the following composition (wt/wt%): glycerol (83.3), methanol (0.01), water (10.0), fatty acids (0.04), salt (6.63), and ash (6.6). The pH was 6.38 and the density was 1.26 g/ml.

Fermentations in shake flasks were performed in 25 ml Pyrex Erlenmeyer flasks (narrow mouth/heavy duty rim, Corning Inc., Corning, NY) filled with 15 ml of 1X MOPS minimal media supplemented with appropriate antibiotics or inducers when needed at the following concentrations: ampicillin (50 μg/ml), kanamycin (50 μg/ml), chloramphenicol (12.5 μg/ml), tetracycline (3.33 μg/ml), and anhydrotetracycline (100 ng/ml). Unless otherwise stated, calcium carbonate (5% wt/wt) was used in all the fermentation flasks to buffer the pH. The flasks (with foam plugs filling the necks) were incubated at 37°C and 200 rpm in an NBS C24 Benchtop Incubator Shaker (New Brunswick Scientific Co., Inc., Edison, NJ). The fermentations were run for 36 hours (unless otherwise stated) at which time the supernatant was collected, the pH measured (UB-10, Denver Instruments Co., Arvada, CO), the optical density taken (Thermo Spectronic Genesys 20, 4001/4, MA, USA), and when necessary cell pellets collected for enzyme activity assays. To determine the optical densities of the cultures in the presence of calcium carbonate, the cultures were allowed to briefly sit in which time the calcium carbonate quickly settled to the bottom.

Prior to use, the cultures (stored as glycerol stocks at −80°C) were streaked onto LB plates and incubated overnight at 37°C. Three colonies were used to inoculate 25-ml flasks containing 5 ml of minimal medium supplemented with 10 g/liter of glycerol, 10 g/liter tryptone, and 5 g/liter yeast extract. The flasks were incubated at 37°C and 150 rpm in an NBS C24 Benchtop Incubator Shaker until an OD_550_ of ~0.7 was reached. An appropriate volume of this actively growing pre-culture was centrifuged, and the pellet was washed and used to inoculate 15 ml of medium in shake flasks (see above) with a target initial optical density at 550 nm of 0.05.

### Analytical methods

The concentration of cell mass, glycerol, organic acids, and ethanol were measured as previously described [45,46]. The enantiomeric purity of lactate was determined enzymatically as previously reported [47]. The reaction mixture (3 ml) for L-lactate determination contained 0.92 ml hydrazine/glycine buffer (0.6 M glycine and 0.5 M hydrazine; pH 9.2), 55 U L-lactate dehydrogenase, 5 mg NAD, and 200 μL of the fermentation sample of interest. D-lactate was measured in a similar mixture by replacing L-lactate dehydrogenase with 15 U of D-lactate dehydrogenase. After addition of the sample, the reaction mixture was incubated at 25°C for 3 hours after which the absorbance at 340 nm was used as a measure of the concentration of D- or L-lactate present.

### Enzyme activities

Cell harvesting and preparation of crude cell extracts for enzyme assays was conducted as described elsewhere [5,7]. Absorbance changes for all assays were monitored in a Biomate 5 spectrophotometer (Thermo Scientific, MA, USA). The linearity of reactions (protein concentration and time) was established for all assays and the nonenzymatic rates were subtracted from the observed initial reaction rates. Enzymatic activities are reported as μmol of substrate per minute per mg of cell protein and represent averages for at least three cell preparations. A protein content of 55% (wt/wt) for *E. coli* cells was assumed in these calculations.

Glycerol kinase and aerobic-glycerol-3-phosphate dehydrogenase activities were assayed as reported previously [15]. Details of the assay can be found elsewhere [15]. The activity of glycerol dehydrogenase in the oxidation of glycerol was measured as previously described [6] with potassium carbonate at pH 9.5 as the buffer. PEP-dependent dihydroxyacetone kinase activity was assayed as previously reported [11]. D-lactate dehydrogenase activity was determined by following the NADH-dependent reduction of pyruvate at 340 nm and 25°C in a 1 ml reaction mixture containing 0.1 M potassium phosphate buffer (pH 7.5), 30 mM sodium pyruvate, 0.33 mM NADH, and 50 μL crude cell extract [48]. The activity of L-lactate dehydrogenase (encoded by *S. bovis ldh*) was determined as described above for D-lactate dehydrogenase but adding fructose 1,6-bisphosphate, an allosteric activator of S. bovis L-LDH [40], to the mixture at a final concentration of 1.2 mM.

### Calculation of fermentation parameters

Data from cell growth, glycerol consumption, and product synthesis were used to calculate volumetric (g/liter/h) and specific rates (g/g cell mass/h) and product yields (g/g glycerol) as previously described [5,11].

## Competing interests

The authors declare that they have no competing interests.

## Authors’ contributions

RG conceived the study. SM, JMC, AM and MDB conducted the experiments. RG, SM, MDB, AM and JMC analyzed the data and prepare the manuscript. All authors read and approved the final manuscript.

## Supplementary Material

Additional file 1: Figure S1 Percent dissolved oxygen (DO) and oxygen transfer rate (OTR) vs. time of wild-type and the final L-lactate biocatalyst. Symbols denote: % DO (■) and OTR (▲). (A) Wild-type MG1655. (B) LA20 *lldD* [pZS-glpK-glpD]. Click here for file
