# Supplementary material for: Efficient synthesis of L-lactic acid from glycerol by metabolically engineered Escherichia coli
Source: Microb Cell Fact. 2013 Jan 25;12:7. doi: 10.1186/1475-2859-12-7 (PMC3616864; doi:10.1186/1475-2859-12-7)
Supplement: Additional file 1: Figure S1 — Percent dissolved oxygen (DO) and oxygen transfer rate (OTR) vs. time of wild-type and the final L-lactate biocatalyst. Symbols denote: % DO (■) and OTR (▲). (A) Wild-type MG1655. (B) LA20 lldD [pZS-glpK-glpD]. [file 1475-2859-12-7-S1.pdf]

SUPPLEMENTAL MATERIALS FOR:

Efficient synthesis of L-lactic acid from glycerol by metabolically engineered *Escherichia coli*

Suman Mazumdar[1], Matthew D. Blankschien[1], James M. Clomburg[1], and Ramon Gonzalez[1,2*]
[1]Department of Chemical and Biomolecular Engineering, Rice University, Houston, Texas and [2]Department of Bioengineering, Rice University, Houston, Texas

[*]To whom correspondence should be addressed:
Department of Chemical and Biomolecular Engineering,
Rice University
6100 Main Street, MS-362
Houston, TX 77005
Phone: (713)-348-4893, Fax: (713) 348-5478
email: Ramon.Gonzalez@rice.edu

Keywords: L-lactic acid, glycerol, metabolic engineering, *Escherichia coli*

To evaluate the dissolved oxygen levels present under our microaerobic conditions, we utilized the DO-166MT-1 Micro Dissolved Oxygen Electrode System as according to manufacturer protocol (Lazar Research Laboratories, Inc., Los Angeles, CA). Flask fermentations were conducted as described in the main text utilizing 40 g/L of refined glycerol, 50 g/L $CaCO_3$, and a starting OD550 of 0.6. The micro electrode was sterilized with 5% w/v formaldehyde, washed with sterile deionized water, and inserted into the media through a hole in the foam plugs made before autoclaving. Figure S1 shows the DO percentages and the volumetric oxygen transfer rates (OTR, see below) vs. time conducting flask fermentations with MG1655 and LA20 *lld* [pZS-glpK-glpD] cultures. Regardless of genotype, the DO levels rapidly decreased from 100% (7.2 mg O_2/L) to near 0% within the first 30 minutes, and, within a period of a couple of hours, fell below the detection limit (microaerobic levels)(Fig. S1). Furthermore, the DO remained below the detection limit well-beyond the time course of our fermentations (□72 hours, data not shown). The volumetric oxygen transfer rate can be calculated as OTR = $k_{L}a$ (C*-C) where C* is the saturated dissolved oxygen level in liquid (7.2 mg O_2/L), C is the actual dissolved measured oxygen concentration, and $k_{L}a$ is the volumetric oxygen transfer coefficient (h^{-1}) (see below). At or near microaerobic levels, C is essentially 0 and the OTR becomes constant (OTR = $k_{L}a$ (C*)), providing a volumetric rate of oxygen uptake equal to the volumetric rate of oxygen transfer. Thus, except for the very initial period right after inoculation, the OTR is constant between cultures for almost the entire course of the fermentation and is determined by the $k_{L}a$ of the flask/conditions. Because of this, the microaerobic conditions can act as an oxygen control, enabling better direct comparison for L-lactate production between genotypes. We evaluated the response time of the micro DO probe used here and found it to be less than a second; therefore the DO values indicate the actual oxygen profiles in the culture. The

k_La for our conditions was then determined by the gassing out method as 19 +/- 1.3 h^{-1}, providing an OTR 137 mg O_2 L^{-1} h^{-1} after the initial period [49].

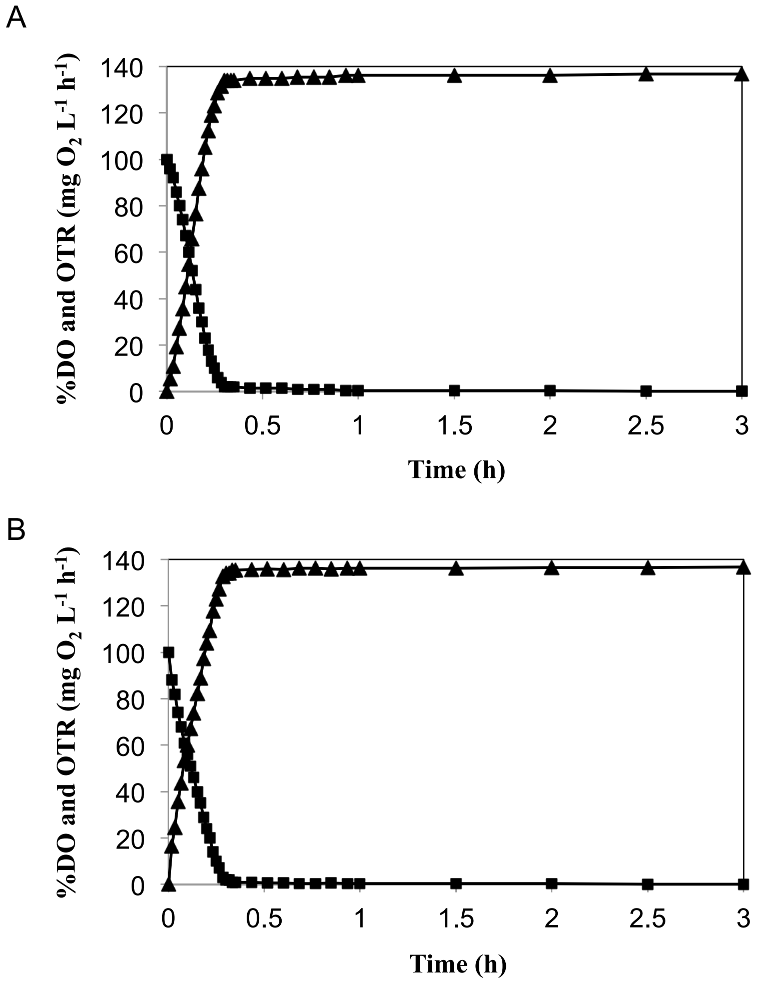

Fig. S1. Percent dissolved oxygen (DO) and oxygen transfer rate (OTR) vs. time of wild-type and the final L-lactate biocatalyst. Symbols denote: % DO (■) and OTR (▲). (A) Wild-type MG1655. (B) LA20 *lld* [pZS-glpK-glpD].

Supplemental References

49. Garcia-Ochoa F, Gomez, E: **Bioreactor scale-up and oxygen transfer rate in microbial processes: An overview.** *Biotech Adv* 2009, **27**:153-176.
